# Advances in the Relationship between Respiratory Viruses and Asthma

**DOI:** 10.3390/jcm12175501

**Published:** 2023-08-24

**Authors:** Sergio de Jesús Romero-Tapia, Crystell Guadalupe Guzmán Priego, Blanca E. Del-Río-Navarro, Manuel Sánchez-Solis

**Affiliations:** 1 Health Sciences Academic Division (DACS), Juarez Autonomous University of Tabasco (UJAT), Villahermosa 86040, Tabasco, Mexico; 2 Cardiometabolism Laboratory, Research Center, Health Sciences Academic Division (DACS), Juarez Autonomous University of Tabasco (UJAT), Villahermosa 86040, Tabasco, Mexico; crystell.guzman@ujat.mx; 3 Hospital Infantil de Mexico Federico Gomez, Mexico City 06780, Mexico; blancadelrionavarro@gmail.com; 4 Paediatric Pulmonology Unit, Virgen de la Arrixaca University Children’s Hospital, University of Murcia, 30120 Murcia, Spain; msolis@um.es; 5 Biomedical Research Institute of Murcia (IMIB), 30120 Murcia, Spain

**Keywords:** asthma, respiratory viruses, rhinovirus, respiratory syncytial virus, asthma exacerbations

## Abstract

Several studies have reported that viral infection is closely associated with the onset, progression, and exacerbation of asthma. The purpose of this review is to summarize the role that viral infections have in the pathogenesis of asthma onset and exacerbations, as well as discuss interrelated protective and risk factors of asthma and current treatment options. Furthermore, we present current knowledge of the innate immunological pathways driving host defense, including changes in the epithelial barrier. In addition, we highlight the importance of the genetics and epigenetics of asthma and virus susceptibility. Moreover, the involvement of virus etiology from bronchiolitis and childhood wheezing to asthma is described. The characterization and mechanisms of action of the respiratory viruses most frequently related to asthma are mentioned.

## 1. Viral Infections as a Risk Factor for Asthma

Asthma is the most common non-communicable disease in children and one of the most frequent chronic diseases in adults, it is generally underestimated or undiagnosed [1,2]. It is currently considered a heterogenous condition that encircles different phenotypes and endotypes with triggering factors such as allergens, changes in the weather, and respiratory viral infections [1,3].

Respiratory viruses may participate as an enabler for asthma exacerbations. The most frequent viruses are rhinovirus (RV), respiratory syncytial virus (RSV), influenza, parainfluenza, adenovirus, coronavirus, human bocavirus, and metapneumovirus. Infections with the aforementioned viruses play a significant role in the occurrence of wheezing, bronchiolitis, and asthma. In children under 12 months of age, RV causes approximately 20 to 40% of bronchiolitis or acute wheezing episodes in the emergency room, which is only second to RSV [4]. Severe bronchiolitis is associated with a high risk of subsequent asthma [5]. Studies with a long-term follow-up described that patients who were presented with respiratory infections by RSV continued to have hyperresponsiveness and persistent airway obstruction up to 30 years later [6].

Results of studies carried out, such as Alsayed et al., show that human rhinovirus (HRV) is classified as the second most frequent cause of acute bronchiolitis in children at risk for asthma. In addition, these results demonstrated the potential utility of the VP4/VP2 region and the VP3/VP1 region for differentiating HRV genotypes [7].

At early ages, infections caused by viruses are considered a risk factor for asthma, in addition to having a family record of asthma or atopy, pre-existing conditions, such as barrier defects and refurbishment, Th2 and 17 inflammation, environmental factors, including high exposure to allergens, tobacco use inside the household, and pollution [5]. Several studies have suggested that the lack of presence or a deficiency in the innate immune response, manifested by low levels of interferons in the epithelial cells in asthma patients, can be associated with a severe asthmatic response. Holt et al. researched the correlation between the innate type 1 and 3 interferon responsiveness (T1/3 IFNs) in postnatal development and susceptibility to viral infections and prevailing wheezing in early ages. The outcome of this study provides evidence of a relationship between the development of T1/3 IFN negative regulation, the susceptibility to low respiratory tract infections, and the subsequent persistent wheezing phenotype associated with asthma in infancy [8]. A meta-analysis was included, where a designated group of children with RV-induced bronchiolitis was shown to have a higher risk of presenting asthma and wheezing in comparison with the counterpart of children with induced RSV bronchiolitis [9].

In children from 3 to 5 years of age with a history of bronchiolitis requiring hospitalization, the presence of asthma was significantly more frequent in children with human metapneumovirus (hMPV) bronchiolitis (odds ratio (OR) = 5.21) vs. RSV bronchiolitis (OR = 4.68) [10]. hMPV infections in children under 2 years of age were considered as risk factors for asthma in later ages [11].

The correlation between viral infections at an early age and the development of asthma and wheezing is possibly related to genetic and environmental factors. For this reason, the genetic factors related to viruses and asthma are described below.

## 2. Genetic Factors, Viral Infections, and Asthma

Knowledge of the genetic mechanisms of asthma has rapidly increased in recent years. Advances in this matter allowed genome-wide association studies (GWAS) to be established, where millions of genetic variants embed the genome [12]. Through GWAS, more than 100 genes/loci associated with asthma as a disease, primarily in childhood, have been identified. The 17q12 locus is the most significant in correlation with asthma in early ages [13].

Premature children are more susceptible to diseases caused by RSV; consequently, biomarkers that act with the innate immune response of children at early ages have been identified, such as a decrease in the expression of the cytokine signaling in the suppressor protein (SOCS2, SOCS3, and SOCS5), as well as a decrease in the production of TNFRSF25 (TNF Receptor Superfamily Member 25) [14].

Epigenome-wide association studies (EWAS) have investigated the association between changes in DNA methylation on asthma development. A large-scale genome-wide meta-analysis of DNA methylation and childhood asthma study has identified new epigenetic variability correlated with asthma in newborn babies and children [15]. When examining the implicated genes, molecules associated with the type-2 immune response were found, including 2q12 (IL1RL1, which codifies the IL-33 receptor), 5q22 (TSLP), 9p24 (IL-33), 5q31 (IL-4, IL-5, IL-13), and other genes that act on inflammatory response, such as SMAD3, BACH2, TLR1/6/10, and STAT6 [16].

Different studies have suggested that the interaction between genes and viral infections has a remarkable role in asthma exacerbations. Studies conducted to assess respiratory infections and illnesses in the COPSAC_2010_ (Copenhagen Prospective Studies on Asthma in Childhood 2010) and COAST (Childhood Origins of Asthma Birth Cohort Study) birth cohorts have shown that the CDHR3 asthma risk allele is specifically associated with RV-C illnesses [17]. In addition, a genome-wide association study identifies CDHR3 as a susceptibility locus for early childhood asthma with severe exacerbations [18].

Due to the genetic diversity of viruses, particularly RV, it is critical to understand genetic variations in an effort to develop effective antiviral or vaccine strategies [19].

In children with early RSV infections and the subsequent development of wheezing and asthma are probably influenced by genetic and environmental factors. Single nucleotide polymorphisms in gene encoding chemokines and cytokines appear to potentially sensitize and increase airway reactivity in both severe RSV bronchiolitis and asthma [20]. Different environmental factors favor early sensitization, such as birth by cesarean section, low birth weight, passive exposure to tobacco smoke [21], and an age of fewer than six months at the time of the infection, the respiratory microbiome, and parental asthma or atopy [22].

## 3. Respiratory Viruses

This section describes the characterization of respiratory viruses associated with asthma including species, family, subfamily, genus, type, subgroup, molecular characterization, protein expression receptor, and capsid.

Throughout human life, acute respiratory infections are the most common disease entity [23]. The World Health Organization program on acute respiratory infections in children points out the importance of recognizing outbreaks and their high contagiousness, morbidity, and mortality, which makes them an emergency for priority healthcare [24]. Knowing the general description of the virome in the respiratory tract starts from the definition of human virome, which consists of all the viruses that colonize the apparatus or systems of the human being. These viruses can infect cells of the human host as well as other microorganisms, such as bacteria, and some can cause disease and others can kill the host asymptomatically [25], so understanding their implications in the development of diseases is a great challenge. Studies in patients with cystic fibrosis have shown that there are different viral populations in each of the regions of the lung [26]. Based on these variations, it is necessary to characterize the viruses that can lodge and develop airway diseases, as summarized in Table 1.

### 3.1. Picornaviridae Family

Their name derives from their small size (pico = small), they are non-segmented single-stranded RNA viruses with positive polarity, and their family includes different genera such as rhinovirus, enterovirus, aphthovirus, and cardiovirus (the last two only affect animals). Their size is 20 to 30 nm. They are naked viruses with icosahedral capsids [31,32] that are resistant to acidic pH, which allows them to have an initial replication in the oropharynx and cross the stomach to colonize the lower digestive tract. Enteroviruses replicate at a temperature of 37 °C and rhinoviruses at 33 °C.

A surprising increase in the number of viruses belonging to the picornavirus family has been identified in the last ten years and distributed globally, all of which retain the characteristic of infecting vertebrate animals of all kinds [33].

### 3.2. Pneumoviridae Family

They are negative-sense RNA viruses that are wrapped in a helical capsid containing the P and M proteins with a diameter of 13.5 nm [34]. Previously, they belonged as a subfamily within paramyxoviridae but in 2016, they were reclassified as a family of two genera: *Metapneumovirus* and *Orthopneumovirus* [35]. Pneumoviruses infect a range of mammalian species, while some members of the metapneumovirus genus may also infect birds.

### 3.3. Metapneumovirus:

They are viruses that infect humans and birds and are divided into four subgroups: A1, A2, B1, and B2.

### 3.4. Orthopneumovirus:

Human, rodent, and bovine viruses are found in this genus. Members of the human orthopneumovirus species are divided into subgroups A1, A2, B1, and B2 [36].

### 3.5. Orthomyxoviridae Family

This family has only one genus: influenza and three species, A, B, and C, whose nomenclature defines the order of their epidemiological and clinical importance [37]. They are oval-shaped negative-sense single-stranded RNA viruses (the oval morphology of the virion is primarily modeled by the M1 subtype of the M protein) with a diameter of 80 to 120 nm, with eight segments for species A and B (that codify ten proteins) and seven segments for C. They have an envelope with two types of surface glycoproteins called neuraminidase (NA) and hemagglutinin (HA) and are arranged over a lipid envelope located on a protein layer (M protein) [38]. The HA binds to the sialoreceptors, penetrates the host cell, and then reaches the nucleus where the messenger RNA (mRNA), which is capable of producing new virions, is replicated. The main activity of the NAs is to release them from the cell [39].

One of the aspects highlighting the influenza virus is the changes in antigenicity, although this is more frequent for type A than type B and is absent for type C. This phenomenon may explain why the flu has been an epidemic disease throughout the years.

### 3.6. Paramyxoviridae Family

This family was described in 1950 and includes the causal agents of common childhood diseases such as measles, mumps, and respiratory diseases [40]. It includes four genera: megamyxovirus, respirovirus, rubulavirus, and morbilivirus. They are RNA viruses with negative polarity with a non-segmented genome that are spherical in shape. Their sizes vary from 150 to 250 nm. Their nucleocapsids exhibit helical symmetry with two nucleocapsid proteins [41].

Parainfluenza viruses are found within the respirovirus genus, which can produce clinical presentations of variable severity, such as mild infections of the upper airways, and are one of the most frequent causes of laryngitis in childhood, which depends both on the type of virus and the host’s characteristics. There are 5 types (1, 2, 3, 4A, and 4B).

Respiratory syncytial virus RSV is a pathogen that causes severe acute respiratory infections in children. It has been reported as the main cause of hospital admissions for respiratory infections in children under one year of age. Its binding to the host is mediated by the G protein, it does not have hemagglutinin, and its fusion protein (F protein) gives it its pathogenicity since it mediates the fusion of the host cell membrane with the viral envelope [42,43].

### 3.7. Adenoviridae Family

Adenoviruses have a linear double-stranded DNA genome, which forms a core associated with polypeptides V and VII, forming 12 subunits with a spherical shape and icosahedral vertex. They are naked viruses and are stable at low pH. Their nucleocapsids are made up of 252 capsomeres that contain their main antigenic determinants, of which 240 are hexons and 12 are pentons [44].

They cause respiratory, urinary, gastrointestinal, and eye infections. The genotypes responsible for clinical presentations change according to their geographical distribution. In North America, the C subgenus predominates, and in South America the B subgroup dominates, and both have a greater presence in winter and spring with a peak incidence between 3 and 5 years of age (for respiratory infections) [45].

### 3.8. Coronaviridae Family

The *coronaviridae* family are enveloped, positive-stranded RNA viruses that can cause disease in birds (coronavirus), mammals (coronavirus, torovirus), and fish (bafinivirus). They have a diameter of 120 to 160 nm, and their virions are spherical. On their membrane, they present large surface projections in the form of peaks (peplomers), which create an image in electron micrographs of the solar corona. The nucleocapsid has helical symmetry and can be released from the virion by the action of detergents [46,47].

*Coronaviridae*, in terms of size, are the largest RNA viruses. Virions can adhere to the host cell surface through the S protein and release their genome through the fusion of their viral envelope with the plasma membrane of the host and start their replication cycle that takes place in the cytoplasm. Four groups of coronaviruses can be distinguished, of which three have been classified as alpha, beta, and gamma. Group number four was recently discovered and was apparently identified as a delta-type bird coronavirus [25,26].

## 4. Pathogenic Mechanisms between the Relation of Infections by Respiratory Viruses and Asthma

Asthma physiopathology is characterized by airway inflammation, hyperresponsiveness, and remodeling, which can trigger lung function decrease due to airway obstruction. RSV or RV respiratory infections set off inflammation and remodeling of the airway tissue, and as a consequence of viral replication and propagation, the integrity of the epithelial barrier may be altered [48].

The process of remodeling the airways in virus-induced asthma is as follows. (1) First, epithelial inflammatory response for viral elimination, which includes the production of cytokines, such as IL-13 and GM-CSF. (2) Second, leukocyte activation, including lymphocytes, neutrophils, eosinophils, mastocytes, and monocytes/macrophages and the production of cytokines and inflammatory factors that derive from all of them. (3) Third, fibroblast and smooth muscle cell proliferation set off by the previous processes. This airway remodeling is generally induced by RV infection, involving chemokines, cytokines, and cellular immunity [49,50].

Considering the phenotypic characteristics, the product of the heterogeneity of asthma, the subtypes have a differentiation in CD4+ Th2 high and Th2 low in the immune cellular expression, and Th2 high conducts the inflammatory process in severe allergic asthma [51].

**Figure 1:**
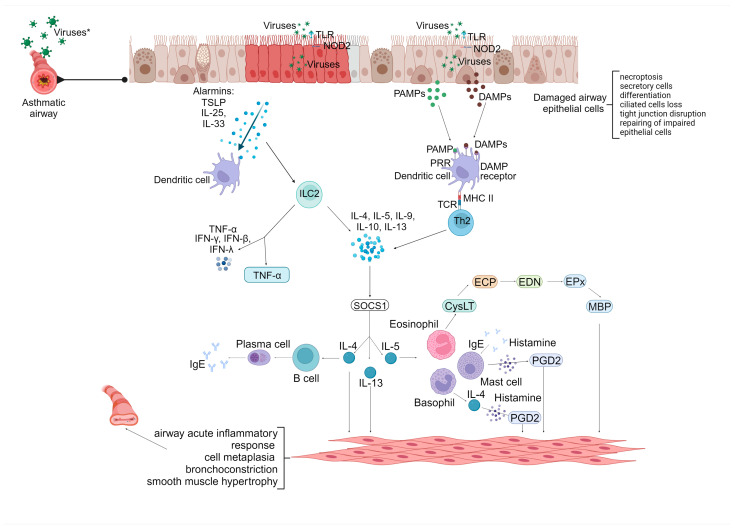
Viral infections and activation of alarmin cytokines in asthmatics. * TSLP: thymic stromal lymphopoietin, TLR: Toll-like receptor, NOD2: nucleotide-binding oligomerization domain containing 2, PAMPs: pathogen-associated molecular patterns, DAMPs: damage-associated molecular patterns, PRR: pathogen recognition receptor, DAMP receptor: damage-associated molecular patterns receptor, MHC II: major histocompatibility complex class II, TCR: T cell receptor, Th2: T helper type 2, ILC2: innate lymphoid cells type 2, TNF-α: tumor necrosis factor-alpha, IFN-γ: interferon-gamma, IFN-β: interferon-beta, IFN-λ: interferon-lambda, SOCS1: suppressor of cytokine signaling-1, IgE: immunoglobulin E, CysLT: cysteinyl-leukotriene, ECP: eosinophil cationic protein, EDN: eosinophil-derived neurotoxin, EPx: eosinophil peroxidase, MBP: major basic protein, PGD2: prostaglandin D2, IL-4: interleukin-4, IL-5: interleukin-5, IL-9: interleukin-9, IL-10: interleukin-10, IL-13: interleukin-13, IL-25: interleukin-25, IL-33: interleukin-33.

The epithelial barrier of the airways is the first place that is affected by viral replication and propagation, which leads to severe damage to the integrity and homeostasis of the airway; it can also result in changes in the allergenic sensitization and unwinding of inflammatory cytokines. Mediators, which are derived from epithelial cells of the airways (AECs), include cytokine alarmins such as IL-25, IL-33, and thymic stromal lymphopoietin (TSLP), which have arisen as crucial elements in the pathogenesis of asthma. When the alarmins of the airway epithelium get released, what happens in asthmatic patients is the over-expression of T2 cytokines, which activates several allergenic mechanisms, including eosinophilic inflammation, IgG switching to IgE, B-cell growth stimulation, goblet cell metaplasia, and the continuous production of mucus [48] (Figure 1).

Once respiratory viruses enter and replicate within airway epithelial cells, invasion depends on interactions with specific receptors, such as intracellular adhesion molecule 1 (ICAM-1), low-density lipoprotein receptors, CDHR3, sialic acids, nucleolin, cell surface integrin, dipeptidyl peptidase 4, and ACE2 [4,49]. There is a greater susceptibility to viral infection in asthma, which originates from the destruction of the epithelium; goblet cell hyperplasia; the positive regulation of growth factors, cytokines, and chemokines; and altered antiviral responses, such as type I IFN production. Direct structural disruption of the airway epithelium caused by injured tight junctions (TJs) and increased epithelial apoptosis results in epithelial leakage. This defective epithelium allows the entry of respiratory pathogens, such as viruses and allergens, into subepithelial and deeper tissues, leading to antigen capture and presentation by DCs [50]. In addition to alarmins, airway epithelial cell responses to viral infection in asthma include proinflammatory cytokines and chemokines, such as eotaxins, RANTES, IL-17, TNF-α, IL-6, IL-8, and IL-1β [52,53].

The presence of human rhinovirus infections in type 2 asthma cytokine alarmins amplifies and upholds the Th2 immune response. At the same time, dendritic cells (DCs) discharge chemokines CC-chemokine ligands (CCL) 17 and CCL22, which promote the recruitment of Th2 cells and ILC2s, paving the way for the spawn of IL-4, IL-5, and IL-13 [54].

Increasing scientific evidence endorses that the physiological manifestation of type 2 asthma is mediated by type 2 cytokines, which are produced as a consequence of the allergen-specific Th2 cells and innate lymphoid cells 2 (ILC2s) activation; these can be associated with damage to the protection against the respiratory virus. ILC2s do not express antigen-specific type-like receptors; on the contrary, they express cytokine, chemokine, lipid mediators, and neuropeptide receptors [55,56]. Diverse studies have suggested that ILC2s are involved in the commencement of asthma and its exacerbations, which is caused by unleashing factors, such as viral infections [51]. Furthermore, it has been proposed that ILC2s are associated with severe asthma [57].

### 4.1. Respiratory Syncytial Virus

The immunological mechanisms before RSV infection start in the nasal epithelial cells, activating the innate response through the activation of type 2 lymphoid cells. Nasal epithelial cells release proinflammatory mediators and recruit immune cells, such as monocytes, macrophages, and DCs. Monocytes induce the release of proinflammatory cytokines, such as TNF-α, IL-1, IL-6, IL-8, IL-10, and IL-18, and promote a Th2-impaired immune response with a parallel decline in lymphocyte maturation and IFN-γ production and a marked release of IL-4 and IL-13. Production of IL25 and 33 by damaged respiratory epithelium stimulates human ILC2; it also promotes the release of IL-4 and IL-13 with a further enhancement of the Th2 immune response. TLR on the monocyte membrane interacts with the RSV F protein and induces increased binding of environmental lipopolysaccharides to the airway epithelium, the activation of mitogen-activated protein kinase (MAPK), and the production of proinflammatory cytokines [58,59].

RSV can establish crucial interactions between the airway neuronal system and the immune response, resulting in long-term airway dysfunction and a predisposition to chronic and persistent airway hyperreactivity and inflammation. These RSV immune system–neuronal pathway interactions are influenced not only by viral factors but also host factors, such as genetic susceptibility, which modifies virus response efficiency, viral replication, and virus-mediated airway injury. The age of primary RSV infection, viral coinfection, and genetic influences can act as effect modifiers [60,61]. Based on bioinformation, new molecular mechanisms have been proposed in RSV infections through the hsa-miR-34b/c-5p/CXCL10 axis, inducing airway inflammation and airway hyperresponsive diseases (AHDs), such as in asthma [62]

RSV was the first virus described in association with the development of asthma in childhood; rhinoviruses and influenza have subsequently been studied. In recent years, hMPB and HboV have been studied, whose mechanisms in asthma are described below [63].

### 4.2. Rhinovirus

RV is the most frequent causative agent of asthma exacerbations. Among the various RV types, RV-A, RV-B, and RV-C draw more attention. Its VP1 protein gives the most antigenicity due to its divergence. Intercellular adhesion molecule 1 (ICAM-1: CD54), cadherine-related family member 3 (CDHR3), or the low-density lipoprotein receptor (LDLR) are cellular receptors of the epithelium [64]. Similar to previous studies, Yuan X et al. found that compared to RSV infection, human rhinovirus (HRV)-C induced relatively low cytokine secretion (except IFN-λ1 and IP-10). Unlike RSV infections, HRV compromises epithelial barrier functions without having a clear cytopathic effect on airway epithelial cells [65,66]. However, the existing mechanisms between the association of rhinovirus, allergic sensitization, and the subsequent development of asthma are not fully elucidated; a causal association is possible between cellular factors that regulate the host’s immune response, airway inflammation and remodeling, and increased production of proinflammatory cytokines and acute rhinovirus infection. RV infection and allergen exposure increase epithelial cells for the production of IL-25 and 33, which promote TH2-type inflammation [12,67]. Additionally, an antiviral response modified by decreased function of type I and III IFNs, an abnormal response of the individual, an increased expression of IgE antibody receptors, or genetic susceptibility due to the expression of CDHR3 in the epithelium, is presented [18].

### 4.3. Metapneumovirus

Other viruses that have been linked to significant respiratory symptoms are metapneumoviruses, which can be severe and cause bronchiolitis. However, reinfections, which typically occur annually, are mild [68]. Human metapneumovirus (hMPV) induces the response of various chemokines and cytokines such as IL-6, IFN-alpha, TNF-alpha, IL-2, and macrophage inflammatory proteins, leading to peribronchiolar and perivascular infiltration and inflammation. The inflammatory process also results in the entry of monocytes and lymphocytes into the endothelium of the airways. These combined responses lead to lung inflammation that causes the respiratory manifestations of cough, mucus production, fever, and dyspnea [69].

The mechanisms involved in acute infection by hMPV and inflammation and respiratory function during infection are different compared to the existing mechanisms in other viruses. In an hMPV infection, alveolar macrophages are activated, producing deleterious effects in the airways; in contrast, RSV infection leads to the depletion of alveolar macrophages, providing protection against their effects [66]. In studies of children with wheezing and hMPV infection, levels of thymic stromal lymphopoietin (TSLP) and IL-4 were higher compared to children without wheezing and hMPV or wheezing and other respiratory viruses. Elevated TSLP levels have been correlated with poor asthma control [70].

### 4.4. Bocavirus

Human bocavirus (HboV) genotype 1 has been isolated frequently in samples of children with wheezing, and coinfection with other respiratory viruses is very high (15–100%) [71]. Studies carried out in children with acute wheezing with RV, HboV, and RV-HBoV coinfection evaluated the proinflammatory cytokine response profile; unlike RV, HBoV infections were not associated with systemic proinflammation or Th2-type response, and in cases with RV-HBoV association, they resulted in a lack of Th2-type immune response [72]. Due to the frequency with which HBoV tends to infect older children and the high rate of coinfection with other viruses, the role of early HBoV infections and the development of asthma has not been clearly established [57].

The association between asthma and HBoV LRT1 infection at early ages requires further prospective studies to confirm the findings up to now [73]. It is essential to carry out studies based on genetic predisposition, demography, etc., in order to establish the correlation between HboV infection in childhood and the development of asthma [74].

### 4.5. Influenza

The influenza virus is one of the viruses often found in patients with asthma exacerbations. However, it is an uncommon cause of acute wheezing in infants [75]. IL-33 has been considered a major inducer of influenza–asthma exacerbations in animal studies. IL-33 production induces IL-13 production in ILC-2 cells, and TH2 cytokine-producing cells promote allergic inflammation in the experimental animal models of asthma and atopic dermatitis [76]. In influenza-infected asthmatic mice, rapid induction of type III IFNs, natural killer cells, and TGF-beta has been documented, indicating a potential mechanism to enhance antiviral immune action against influenza in asthmatics [77].

### 4.6. Coronavirus

SARS-CoV-2 is a member of the Betacoronavirus genus, belongs to the Coronaviridae family, and is related to SARS-CoV and Middle East respiratory syndrome [78]. In infected cells, the SARS-CoV2 S protein binds to the main cellular receptor, angiotensin-converting enzyme 2 (ACE2), and the host serine protease TMPRSS2 is important for proteolytic priming of the S protein for receptor interactions and entry [79]. In particular, TLRs, RLRs, NLRs, and inflammasomes have been shown to activate their signaling pathways in response to SARS-CoV-2 and induce cytokine production [80]. PRR signaling engaged by SARS-CoV-2 induces the concurrent release of both IFNs and other proinflammatory cytokines, including IL-1 beta, IL-6, TNF, IL-12, INF beta, and IL-17, among others [81].

Because the ACE2 receptor has a central role in SARS-CoV-2 infection, it is closely related to the severity of COVID-19. The ACE2 expression gradient is mainly expressed in the upper airway epithelium, implying a higher susceptibility of upper airway cells to SARS-CoV-2 infection [82]. In the Urban Environment and Childhood Asthma (URECA) cohort, children at a high risk of asthma were prospectively followed based on a family history of atopy. In children with asthma, moderate allergic sensitivity and high allergic sensitivity were associated with progressively greater reductions in ACE2 expression compared to children with asthma but no/minimal allergic sensitivity. These results suggest a potential mechanism for reducing the severity of the response to COVID-19 in patients with asthma and respiratory allergies [83].

Jayavelu et al. have shown evidence that in patients with type 2 inflammation, the expression of ACE2 is suppressed and the expression of TMPRSS2 in the epithelial cells of the airways is increased in the presence of asthma and atopy [84]. In asthmatic patients with high T2 inflammation, Kimura et al. demonstrated decreased mRNA expression of ACE2 in bronchial epithelial cells. IL-13 modulates ACE2 and TMPRSS2 expression [85]. IL-13 downregulates the expression of the angiotensin-converting enzyme 2 (ACE2) host cell entry receptor of SARS-CoV-2 in airway epithelial cells, and decreased levels of ACE2 transcripts are associated with allergic diseases [83]. Eosinophil activation is dependent on Th2 cell-associated cytokines, primarily IL-5. Eosinophilia has been found to be negatively associated with susceptibility to COVID-19, with eosinopenia being a biomarker of severe disease. These mechanisms associated with asthma type 2 inflammation may reduce the risk of presenting severe COVID-19 [83]. Patients with type 2 asthma have a decreased risk of presenting severe disease from COVID-19 due to the complexity of both conditions. A greater number of investigations are required to understand the association between SARS-CoV-2 and asthma [86,87].

## 5. Viral Infections and Asthma Exacerbations

Usually, exacerbations occur in response to diverse agents such as viral infections of the upper respiratory tract, pollen, or pollutants associated with poor adhesion to medication (controller medication) [1]. Oxidative stress, pollutants (tobacco exposure) upsurge, and lifestyle are determinant factors for the lack of control of asthma in children [88].

**Figure 2:**
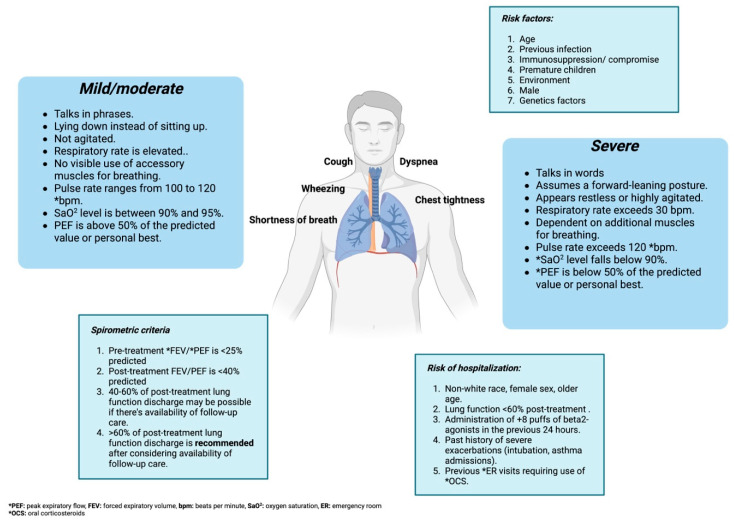
A comprehensive assessment of a patient with asthma exacerbation and viral infection.

Asthmatic exacerbation caused by viral infections can occur in patients with a pre-existing asthma diagnosis or can also be its first form of presentation. Integral evaluation of the patient who presents an exacerbation must include risk factors for asthma-related mortality, criteria for assessing severity, and risk factors for hospitalization [1] (Figure 2).

The comprehensive assessment of a patient with exacerbation should include the identification of risk factors for asthma-related mortality, clinical criteria to evaluate the severity of the condition, and risk factors for hospitalization [1] (Figure 2).

Viral infections contributed to more than two-thirds of asthmatic exacerbations in children and more than half of affected adults. Other viruses that are associated with acute asthma, aside from RV, include RSV, enterovirus, influenza A and B, parainfluenza virus, adenovirus, and coronavirus. It has been substantiated that viral infections have been around for about 50–80% of asthma exacerbations, and RV has been detected in close to 50–80% of patients [89].

Various factors determine the severity of viral infections in asthmatic patients, including host-related factors, virus-related factors, and gene-related factors. Regarding host-related factors, several reports suggest that patients with asthma are more susceptible to viruses, including RV, and their symptoms are easily exacerbated by RV infections [89]. Accumulated evidence from numerous studies has reported that the innate antiviral immune response (including type I and type III interferons) in asthma patients is less effective or deficient, leading to the conclusion that epithelial innate immunity is a crucial determinant of severe disease during RV-induced asthma exacerbation. Through the induction of gene expression by interferons (type I, II, and III) and interferon response factors, TLR signaling, NK-kB activation, and STAT-1 activation, it has been proposed that the propensity for viral exacerbation of asthma and COPD is related to a delayed (rather than deficient) expression of innate epithelial cells and antiviral immune genes, which resulted in a delayed host immune response and increased persistent inflammation [55].

Recently, the importance of virus-related factors in asthma exacerbations has been highlighted. Infections caused by RSV are associated with the induction of the Th-2 immune response. CX3CR1, the receptor for RSV G protein, and its ligand CX3CL1 exacerbate allergic immune response. However, compared to RV infection cases, the concentrations of IL-5 in serum during RSV infection do not increase; this suggests that the degree of type-2 deviation may be lower than in RV infection [89].

Recent studies have been carried out to analyze the mechanisms of response to RV infections in asthma patients and experimental in vitro studies. They have established that asthmatic patients with RV infections promote excessive activation of RIG-1 inflammasome with subsequent decreased response to type I/III interferons, leading to a delayed and prolonged resolution of epithelial inflammation [90].

A prospective study conducted by Dinwiddie et al. reported that uncontrolled asthma patients with viral infections had more acute symptoms during exacerbations, especially in those with allergies. These data imply a possible synergy between viral infection and allergy in subjects with uncontrolled asthma, considering acute asthma symptoms and the evaluation of biomarkers during asthma exacerbation [91].

## 6. Current Therapeutics for Viral Infection in Asthma

Despite viral infections being the most common causes of asthma exacerbations, the prevention and treatment of viral infections that induce asthma remain without fully satisfactory treatment. Exacerbations occur as part of the spectrum of severe asthma and constitute a significant burden on healthcare systems and affected patients [4].

**Figure 3:**
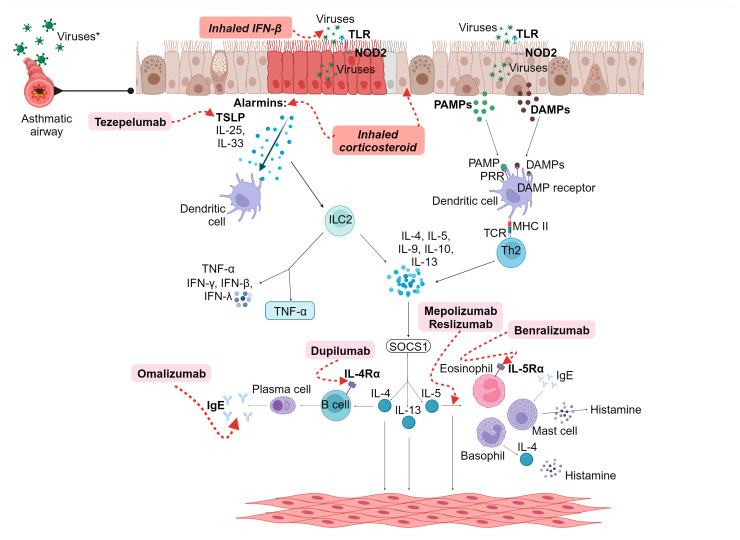
Current therapeutics for viral infection in severe asthma. * TLR: Toll-like receptor, NOD2: nucleotide-binding oligomerization domain containing 2, TSLP: thymic stromal lymphopoietin, PAMPs: pathogen-associated molecular patterns, DAMPs: damage-associated molecular patterns, PRR: pathogen recognition receptor, DAMP receptor: damage-associated molecular patterns receptor, MHC II: major histocompatibility complex class II, TCR: T cell receptor, Th2: T helper type 2, ILC2: innate lymphoid cell type 2, TNF-α: tumor necrosis factor-alpha, IFN-γ: interferon-gamma, IFN-β: interferon-beta, IFN-λ: interferon-lambda, IL-4: interleukin-4, IL-5: interleukin-5, IL-9: interleukin-9, IL-10: interleukin-10, IL-13: interleukin-13, IL-25: interleukin-25, IL-33: interleukin-33, SOCS1: suppressor of cytokine signaling-1, IgE: immunoglobulin E, IL-4Rα: interleukin-4 receptor alpha, IL-5Rα: interleukin-5 receptor alpha.

Among the medications included for preventing and treating viral-induced asthma exacerbations are inhaled corticosteroids (ICS) and the new generation of anti-type 2 biologicals, which have shown impressive efficacy in reducing the rate of acute asthma exacerbations. They may also be effective during viral infections by improving the antiviral response [4]. The mechanisms of action of these new-generation biological medications act on different crucial points of bronchial inflammation, inhibiting various cytokines and/or their specific receptors, thus effectively controlling symptoms and reducing the use of systemic steroids. Examples of these biologicals include omalizumab targeting IgE, mepolizumab and reslizumab targeting IL-5, benralizumab targeting the interleukin 5 alpha receptor, and dupilumab targeting IL-4 and IL-13. Additionally, recent additions to this group of medications include itepekimab targeting IL-33, tezepelumab, a monoclonal antibody blocking thymic stromal lymphopoietin, and astegolimab as an anti-TSLP (thymic stromal lymphopoietin) 2 agent [92] (Figure 3).

Among the medications used for viral-induced asthma exacerbations are anti-type biologicals, such as tezepelumab, which blocks TSL, and Astegolimab, as an anti-TSLP 2 agent. They have been used to decrease IL-5 and IL-13 serum concentrations and eosinophilic inflammation. Omalizumab works by blocking IgE, which reduces susceptibility to RV infections during asthma exacerbations. Dupilumab targets IL-4Rα and blocks the effects of IL-4 and IL-13, which contribute to the reduction in different markers of T2 inflammation, including FENO, IgE, periostin, and eotaxin-3, among others. Mepolizumab and reslizumab bind to free IL-5, blocking it and its receptor and leading to a decrease in eosinophilic activation. They act as essential mediators for suppressing eosinophil activity. Benralizumab directly targets IL-5Rα, effectively countering its activity. In addition, inhaled corticosteroids act on inflammatory cells within the airway epithelium, suppressing the production and release of proinflammatory mediators. Inhaled IFN- β helps inhibit viral replication by interfering with the virus’s life cycle and blocking its ability to infect and propagate within cells [93].

### 6.1. Inhaled Corticosteroids (ICS)

The use of ICS as maintenance therapy that is fundamental in asthma treatment is effective in reducing the risk of future asthma exacerbations caused by the virus, along with decreasing the risk due to the use of long-acting β-2 agonists [1]. Cochrane Database review signals that is implausible for a reduction in the need for oral steroids if the dosage increases in adults and children with mild to moderate asthma in the presence of the first couple of clinical manifestations of an exacerbation [94]. Several studies have proven that asthma exacerbations have different spectrums according to inflammatory endotypes. There is evidence that upholds that the patient with eosinophilic inflammatory asthma has a good response to steroids. On the other hand, non-eosinophilic inflammation has a mild response [95].

### 6.2. Omalizumab

According to the PROSE (Preventative Omalizumab or Step-up Therapy for Severe Fall Exacerbations) study, 478 children (6–17 years old) with allergic asthma have been examined; omalizumab treatment decreased the effects of RV infections and risks of RV-induced diseases during asthma exacerbations. The obtained results provide evidence that the blockage of IgE diminishes the susceptibility of RV infections in asthma exacerbations [96]. Due to exacerbations induced by viral infections, the incidence rate is higher in the fall season; a double placebo-controlled, multicentric clinical trial was conducted among inner-city asthmatic children aged 6–17 years old with one or more recent exacerbations. The study found that a 4–6-week pre-seasonal treatment of omalizumab significantly diminished the exacerbation incidence rate. Furthermore, omalizumab improves the INF-α response to rhinovirus. This improvement is correlated to fewer exacerbations [97].

A meta-analysis in 2020, which includes randomized controlled trial (RCT) studies, was conducted on children and teenagers with asthma and compared to the efficacy and safety of omalizumab. The obtained data suggest that this agent reduces the asthma exacerbation rate during a 30-week+ period treatment program and has a low incidence of adverse effects [98].

### 6.3. Mepolizumab

The use of mepolizumab is approved for patients who have severe asthma with an eosinophilic phenotype. A Gupta et al. study concluded that following a long-term secure profile, pharmacodynamics, and effectiveness data, a positive risk–benefit factor for mepolizumab in children with severe asthma and an eosinophilic phenotype were found. This may be similar to statistics of other authors with teenagers and adults as a subject of study [99]. Other studies suggest that treatment with mepolizumab is associated with a reduction in the exacerbation’s frequency and ICS usage, and cost–benefit factors are tied to the relationship between asthma exacerbations in patients with severe asthma [100].

### 6.4. Dupilumab

An IL-4Rα targeted biologic agent, blocking the effects of IL-4 and IL-13, contributes to the reduction in different markers from T2 inflammation, including FENO, IgE, periostin, and eotaxin-3, among others. Dupilumab could also improve antiviral immune response in patients with severe T2 asthma on account of IL-4 and IL-13 by damaging the production of viral-induced IFN that expresses TLR3 [4,101].

Through a systematic review and meta-analysis, they were addressed to evaluate the efficiency and security of dupilumab on patients with mild/severe asthma, including thirteen trials. It has been determined that dupilumab improves in a significant way the FEV in 1 s, the nitric oxide exhaled fraction, and IgE levels from 12 to 24 weeks of treatment (*p* < 0.05) [102].

### 6.5. Benralizumab

A monoclonal antibody that acts directly against the IL-5Rα chain, a key cytokine in eosinophil maturation and differentiation, resulted in a quick eosinophilic reduction through cellular cytotoxicity that is reliant on antibodies [91]. Benralizumab is indicated in patients who are 18 years old or older with severe eosinophilic asthma. By using bronchoscopy, a study demonstrated that an IV dose and multiple subcutaneous doses of benralizumab reduced the eosinophilic count in the bone marrow and peripheric blood. Deployment of agents like benralizumab on severe eosinophilic asthma can be a reasonable strategy to decrease viral-induced asthma exacerbations [103].

Recent studies have suggested that the presence of eosinophilic inflammation could be a risk factor for asthma exacerbations in correlation with viruses. The damaged epithelial cells are a consequence of the eosinophil’s pellets, which can derive from a major protein base (MPB) that increases susceptibility to RV infections. Furthermore, eosinophils can suppress interferon expressions (IFNs) and antiviral cytokines, including IFN-λ from epithelial cells, by resulting in an increase in the amount of RV [104].

### 6.6. Anti-Thymic Stromal Lymphopoietin mAb (Tezepelumab)

Approved to use in patients over 12 years old with severe asthma, it disables the interaction between the anti-thymic stromal lymphopoietin receptor and differs from other biological agents in which it is not necessary to prove eosinophilia or a T2 high phenotype [103].

Tezepelumab could reduce exacerbations in patients with severe asthma. Moreover, it could also decrease IL-5 and IL-13 serum concentrations and eosinophilic inflammation. Nevertheless, it has been assumed that tezepelumab can suppress the viral induction of ILC2 and eosinophilic inflammation in animals and the anti-TSLP Ab effect over the innate immune response that is viral-induced. The ILC2 activation in asthma patients must be examined in further investigations [57].

## 7. Directions for Future Research

Viral respiratory infections that give rise to asthma exacerbations, mainly rhinovirus, are a cause of morbidity and mortality at increased levels, in some cases with ICS resistance or a detrimental outcome. The specific mechanisms in Th1 or Th2 differential responses in atopic asthma patients or virus-induced exacerbations (Th17 or Th17/Th2 response), with the proinflammatory and suppressor Treg cell participation, suggest that new asthma-related paradigms are reasons for forthcoming investigations [105].

Several opportunities can be used to recast the diagnosis and asthma treatment and its associated exacerbations, such as viral infections. Powerful tools and omics technologies are now available for this purpose, as well as specific monoclonals. It is a priority that these tools will be utilized rationally [106].

As part of the primary prevention of RSV infections, especially in neonates and younger infants, the bivalent RSV preF vaccine has been investigated. This vaccine is based on the antigenic subgroups A and B of RSV and has been investigated with the MATISSE study group, vaccinating mothers during their pregnancy and intending to produce maternal antibodies with passive immunity for newborns obtaining protection against RSV. This study establishes that the RSV preF vaccine, administered during pregnancy, has been effective against severe RSV-associated lower respiratory tract infections in infants [106].

It is necessary to carry out a greater number of research studies that allow us to deepen our knowledge of the immunological mechanisms of respiratory viruses and their relationship with asthma.

## 8. Conclusions

The close association between viral infections and the origin and exacerbation of asthma, mainly in pediatric ages, has been widely documented by several studies. Current knowledge on the mechanisms of action of viruses in the development of airway inflammation and the involvement of the innate immune response, especially in the activation of ILC2s and epithelial cell-derived cytokines called alarmins, including IL-25, IL-33, and TSLP;, and the timely inhibition of the epithelial RIG-1 inflammasome, open a window of opportunity for the use of new drugs, mainly biologics, in the prevention and treatment of asthma exacerbations due to viral infections.

The complexity and heterogeneity of asthma have led to the use of new omics technologies that allow a better understanding of the pathogenesis of this disease.

## Figures and Tables

**Table 1:** Characterization of respiratory viruses related to asthma.

Species	Family	Subfamily	Genus	Type	Subgroup	Gene	Molecular Characterization	Protein Expression–Receptor	Capsid
Rhinovirus	Picornaviridae		Enterovirus	RNA	HRV-A, HRV-B, HRV-C	IFIH1	The surface of the rhinovirus has shaped-like canyons that provide an *attachment site for its receptors on the surface of susceptible target cells. * Attachment site: ICAM-1.	*VP1*—binding site function to the receptors of the surface. *VP1*, *VP2*, *VP3*—antigenic diversity. *VP4*—the ssRNA is anchored to its core capsid. Receptor * LDRL—attachment and internalization. * ICAM-1RV-A, RV-B * CDHR3-RV-C.	Its viral capsid encircles the RNA genome, composed of 60 identical copies of its structural proteins. [27].
Respiratory syncytial virus	Pneumoviridae	Pneumovirinae	Orthopneumovirus/Pneumovirus	RNA	A and B	M, M1, M2, M2-1 NS1, NS2 P	RSV virions consist of the structure of a nucleocapsid packaged in a lipid envelope matter that is derived from the host cell plasma membrane. These spherical particles are 100-350 nm in diameter.	F, G glycoproteins—viral neutralization antigens. Signals a cleaved. N—terminal pattern. F—viral penetration and syncytium emergence pathway. Receptor CX3CR1, nucleolin, EGFR, IFG1R, HSPGs.	Made from a polymerase protein, nucleoprotein, and a phosphoprotein.
Metapneumovirus	Pneumoviridae	Pneumovirinae	Metapneumovirus	RNA	A (A1, *A2*) and B (B1, *B2*)	N, P, M, F, M2, SH, G, L	The size of its virions can go from 150–600 nm with a spike-like envelope. Its *-ssRNA genome is suspected to be the starring cause of the main lower respiratory tract illness among children.	Glycoproteins. F—fusion protein. * SH protein—small hydrophobic protein. G—attachment protein. Receptor RGD-binding integrins. αvβ1 integrin.	The metapneumovirus forms a left-handed helical nucleocapsid to shelter the RNA genome as well as protect its RNA from nucleases. [28].
Human bocavirus	Parvoviridae	Parvovirinae	Bocavirus	DNA	HBoV1, HBoV2, HBoV3, HBoV4	RNA Pol III	Bocaparvoviruses have both a proximal and a distal *polyadenylation site within their promoters. This transcribes exclusively the (pre-) mRNA precursor. The NP1 expression in bocavirus has an open reading action that is in its genome center. *Polyadenylation sites: (pA)p and (pA)d.	Non-structural proteins *NS1*, *NS1-70*, *NS2*, *NS3*, *NS4*, and *NP1*. Structural proteins *VP1*, *VP2*, and *VP3*.	The HBoV capsid has a protective coat function for the genome, thus logging on to start the infection process.
Adenovirus	Adenoviridae		Mastadenovirus	DNA	HAdV A, HAdV B HAdV C HAdV D, HAdV E, HAdV F, HAdV G	E1, E3 dl309 (mutant)	There are 49 serological types of adenoviruses that have the capacity to infect humans. This commonly causes upper and lower (acute) respiratory infections. These infections can lead to a severe or fatal outcome in immunocompetent/immunocompromised patients. Different HAdVs groups own slight changes to their tropisms, correlating with other clinical manifestations.	Protein V—viral DNA to the cell nucleus pathway. Protein IX—transcriptional activator. p32K—unique structural protein.	Built out of an icosahedral capsid made up of 252 capsomeres. A double-stranded DNA virus of 70 to 90 in size.
Coronavirus	Coronaviridae	Coronavirinae	Alphacoronavirus, Betacoronavirus, Gammacoronavirus, Deltacoronavirus	RNA	*Betacoronavirus—A*, *B*, *C*, *D*	ORF1ab N gene	This virus has a unique way of operating its genome expression. The replication merely stands on the frameshifting of its ribosome during the translation process of the genome and the progeny virions assembly.	* Nsp1	The coronavirus forms helical capsids, which are resistant to RNase because of the binding properties of the N protein. [29].
Parainfluenza 1,3	Paramyxoviridae	Paramyxovirinae	Respirovirus	RNA	HPIV-4a, HPIV-4b		Parainfluenza virions have pleomorphic properties, in which the diameter ranges from 150 to 200 μm. They have been grouped into four serotypes, where they are seemingly divided into respirovirus, which includes HPIV-1 and HPIV-3, and rubulavirus, which HPIV-2 and HPIV-4 are a part.	N—Nucleocapsid protein. P—Phosphoprotein. F—Fusion glycoprotein. M—Matrix protein. HN—Hemagglutinin neuraminidase glycoprotein. L—RNA polymerase. Receptor α2-3-linked * SAs with sulfated sialyl-Lewis motif α2-8-linked * SAs.	The PIV5-N nucleocapsid ring encapsulates a nuclease-resistant 78-nt RNA strand.
Parainfluenza 2,4	Paramyxoviridae	Paramyxovirinae	Rubulavirus						
Influenza	Orthomyxovirus		Orthomyxovirus	RNA	A, B, C	M gene	RNA genomes with a negative segmented RNA strand are one of the principles of the formation of the virion. It requires the possession of an RNA-dependent RNA polymerase native from a viral origin for replication. The drift and shift of the virus has antigenic properties that are being enabled by the genome structure of the *influenza* virus. [30].	PB1, PB2, PA. Receptor. A2,3- and α2,6 receptors.	The capsid is surrounded by the M1 matrix protein made out of a lipid bilayer where hemagglutinin, neuraminidase, and M2 proteins are tailored.
Enterovirus	Picornaviridae		Enterovirus	RNA	Coxsackievirus A 1-22, 24 Rhinovirus Poliovirus Echovirus 1–7, 9, 11–27, 29–34		The non-enveloped virus, such as enterovirus, has approximately 7500 nucleotides, containing a single open reading frame that encodes polyproteins that are then processed to yield the structural proteins.	*Precursor proteins* (*polyproteins*). P1 - *VP1*, *VP2*, *VP3*—*capsid surface*. - *VP4*—*within the capsid*. P2, P3.	They have a non-enveloped icosahedral * +ssRNA capsid. Their replication takes place in the cytoplasm.

* +ssRNA: positive single-stranded RNA, -ssRNA: negative single-stranded RNA, ICAM-1: intercellular adhesion molecule 1, SA: sialic acid, SH: sulfhydric, LDRL: low-density lipoprotein receptor, CDHR3: cadherin-related family member 3, CX3CR1: CX3C chemokine receptor 1, EGFR: epidermal growth factor, IGF1R: insulin-like growth factor-1 receptor, HSPGs: heparan sulfate proteoglycans, Nps1: non-structural protein-1.

## Data Availability

Not applicable.

## Abbreviations

RV	Rhinovirus
RSV	Respiratory syncytial virus
Th2	T helper type 2
ILC2	Innate lymphoid cells type 2
IgE	Immunoglobulin E
TSLP	Thymic stromal lymphopoietin
NOD2	Nucleotide-binding oligomerization domain containing 2
TLR	Toll-like receptor
TNF-α	Tumor necrosis factor-alpha
IFN	Interferon
hMPV	Human metapneumovirus
